# Surgical resection for small bowel obstruction due to right ovarian metastasis after ileocecal resection for cecal cancer: A case report

**DOI:** 10.1002/ccr3.5521

**Published:** 2022-03-01

**Authors:** Naohiko Otsuka, Kimiyuki Shirayama

**Affiliations:** ^1^ Department of Surgery Fujiwara Memorial Hospital Katagami Japan

**Keywords:** colorectal cancer, oophorectomy, ovarian metastasis, palliative surgery

## Abstract

Although oophorectomy for ovarian metastasis from colorectal cancer is encouraged to improve the prognosis, that is also performed to relieve the symptom such as abdominal distention. We report a surgical case of intestinal obstruction due to ovarian metastasis after ileocecal resection for cecal cancer diagnosed at 77 years old.

## Graphical abstract

For small bowel obstruction due to right ovarian metastasis from cecal cancer, right oophorectomy and partial resection of the small intestine were done.

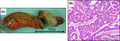

## 1 INTRODUCTION

Metastatic ovarian tumors account for 10%–15% of malignant ovarian tumors, and most of them originate from the cancers of the gastrointestinal tract, especially gastric cancer, or from breast cancer.^1^ Although rare, the incidence of ovarian metastasis from colorectal cancer has increased recently with the increase in patients with colorectal cancer.^2^, ^3^ Some studies have reported that oophorectomy for ovarian metastasis from colorectal cancer should be performed since it seems to contribute to prolonged survival. On the contrary, palliative surgery is also performed to relieve the symptom such as abdominal distention caused by metastatic ovarian tumor.

There has not been a case report so far that describes intestinal obstruction due to ovarian metastasis from colorectal cancer, and we report the first case of small bowel obstruction due to right ovarian metastasis after ileocecal resection for cecal cancer, treated with right oophorectomy and partial resection of the small intestine.

## 2 CASE REPORT

**FIGURE 1:**
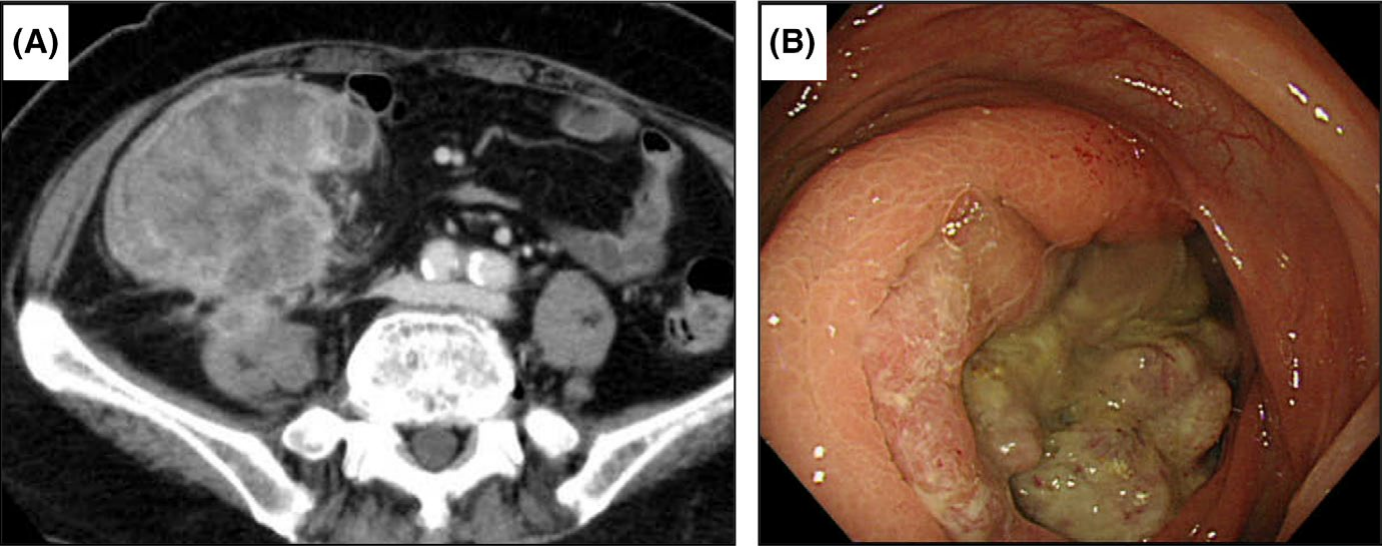
(A) Computed tomography scan showed a tumor, measuring 9 × 8 cm, in the ascending colon, suspectedly infiltrating the psoas major muscle. (B) Colonoscopy revealed a circumferential tumor in the ascending colon

**FIGURE 2:**
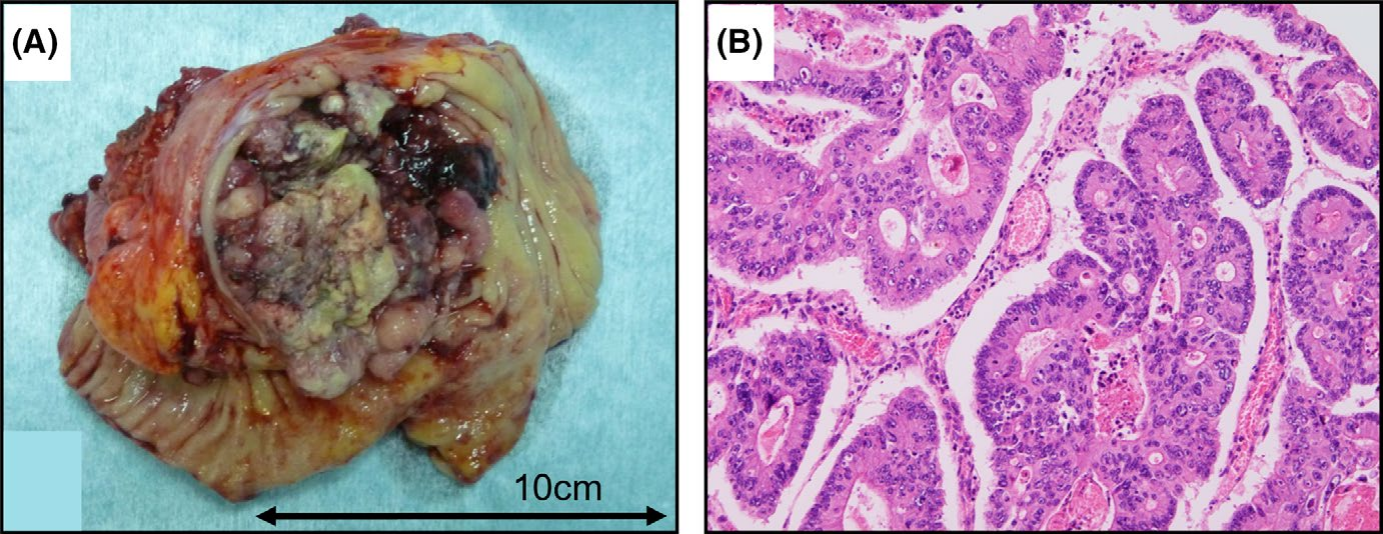
(A) Size of the primary lesion was 10 × 8 cm in the resected specimen. (B) Histological section of the primary lesion demonstrated well‐differentiated tubular adenocarcinoma (hematoxylin and eosin staining; ×100)

A 77‐year‐old woman was referred to our hospital for fatigue and a palpable right lower quadrant mass. Blood tests showed mild anemia (hemoglobin, 9.2 g/dl), and the tumor markers were within normal limits (carcinoembryonic antigen, 2.20 ng/ml and carbohydrate antigen 19–9, 7.0 U/ml). A computed tomography (CT) scan showed a large tumor, measuring 9 × 8 × 8 cm, ranging from the cecum to the ascending colon, suspectedly infiltrating the retroperitoneal space (Figure 1A). A circumferential tumor was found on colonoscopy (Figure 1B), and the endoscopic biopsy revealed well‐differentiated tubular adenocarcinoma (tub1). She underwent the operation. Although there was no peritoneal dissemination and ascites, the cancer infiltrated the right abdominal wall and retroperitoneal space, including the psoas major muscle. Since the tumor was located mainly at the cecum, we judged that right hemicolectomy was unnecessary to secure the distal margin. Ileocecal resection with D3 lymph node dissection was performed. Unfortunately, cancer remnants were detected in the retroperitoneal space, and pathological findings revealed a positive radial margin. The pathological diagnosis was C, Type3, 10 × 8 cm, tub1, T4b (psoas major muscle), N0, M0, StageIIc, PM0, DM0, RM1, R1, and CurB, according to the 9th Edition of the Japanese Classification of Colorectal, Appendiceal, and Anal Carcinoma (Figure 2).

**FIGURE 3:**
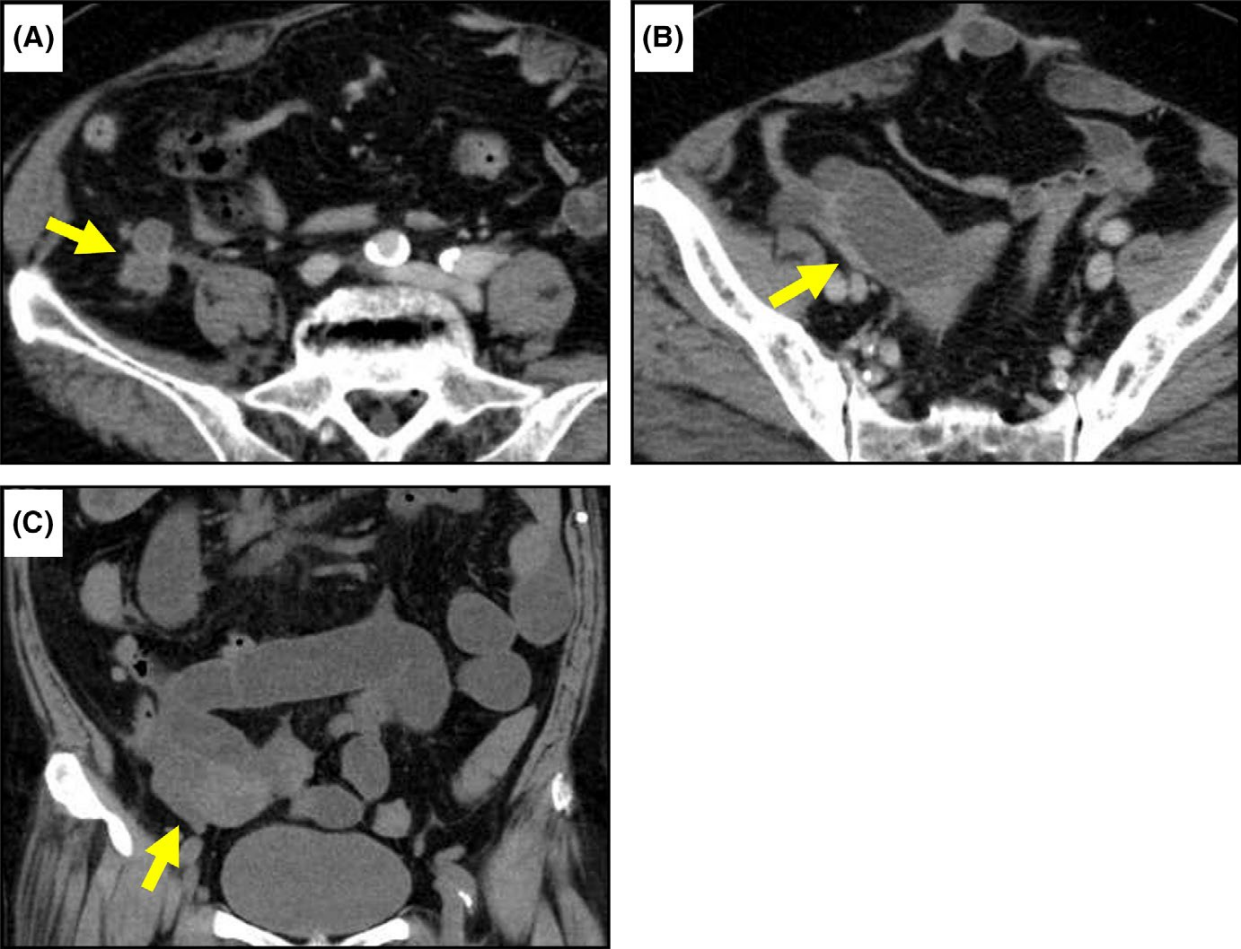
Follow‐up computed tomography (CT) scan showed local recurrence (A) and an enlarged right ovary (B). (C) The patient reported frequent vomiting, and a CT scan showed small bowel obstruction, likely caused by the ovarian tumor, directly infiltrating the small intestine (The arrow indicates the ovarian tumor)

**FIGURE 4:**
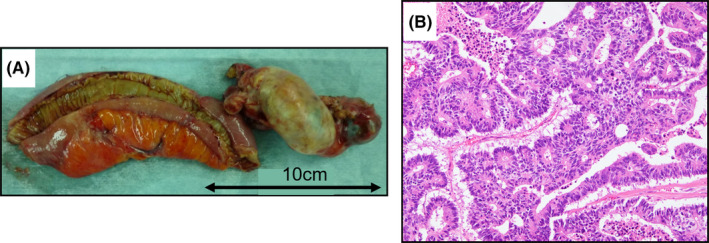
(A) Ovarian tumor, measuring 6 × 4 × 3 cm, and the small intestine were resected. (B) Histological diagnosis of the ovarian tumor was well‐differentiated tubular adenocarcinoma (hematoxylin and eosin staining; ×100)

**FIGURE 5:**
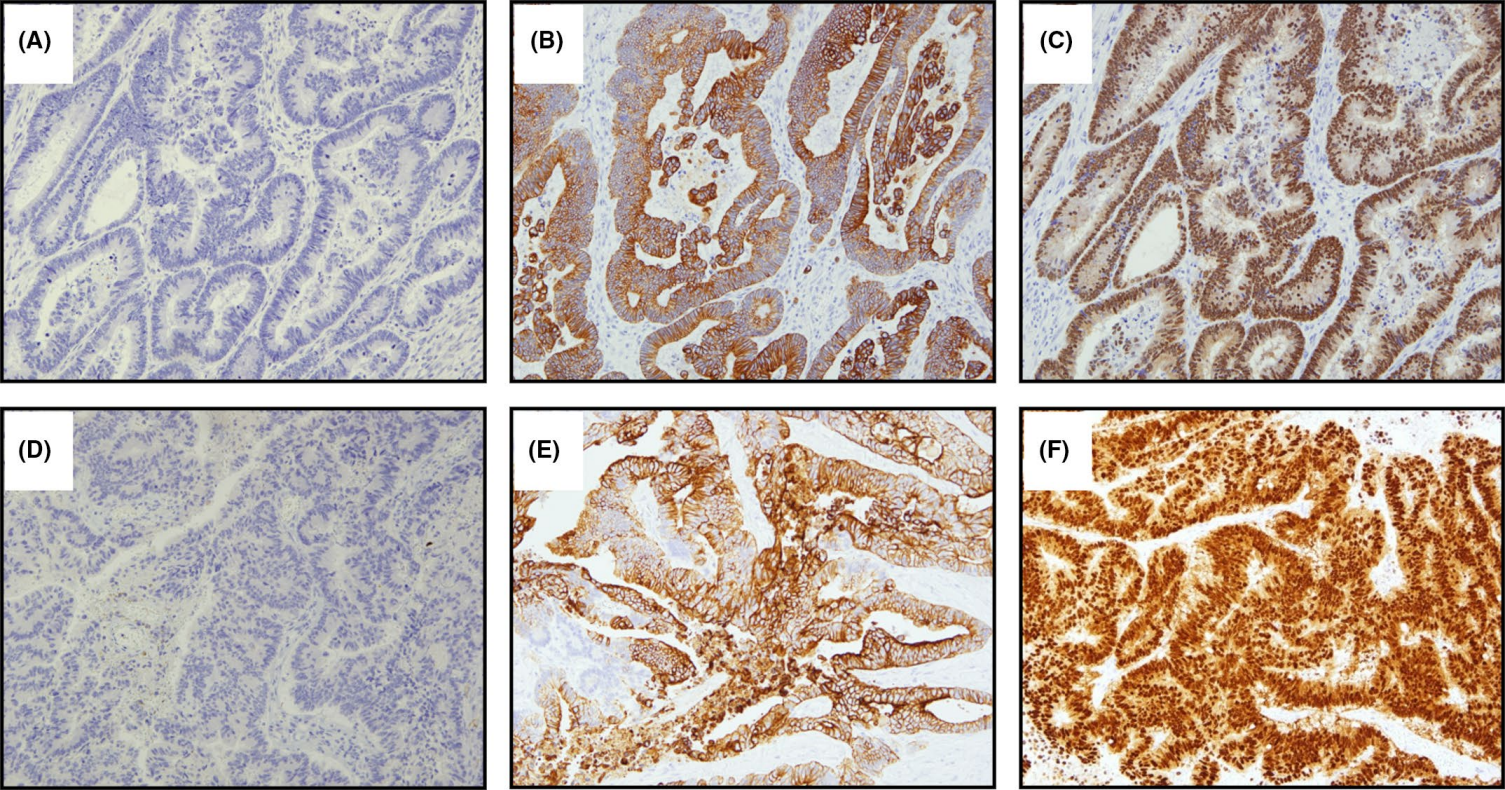
On immunohistochemical staining (×100), the primary lesion was negative for cytokeratin 7 (CK7) (A), and positive for CK20 (B) and caudal‐related homeobox 2 (CDX2) (C). The results of the ovarian tumor were consistent with those of the primary lesion (D–F)

Capecitabine was administered as adjuvant chemotherapy, but the treatment was discontinued 4 months later due to inferior vena cava thrombosis. After chemotherapy discontinuation, local recurrence occurred (Figure 3A). Follow‐up CT scans revealed a gradually enlarging right ovary, suggesting ovarian metastasis from cecal cancer (Figure 3B). Therefore, tegafur, gimeracil, and oteracil potassium (S‐1) were started 27 months after the primary surgery. The follow‐up CT scan documented the shrinkage of the ovarian tumor. However, she was admitted for complaints of vomiting 35 months after the primary surgery. A CT scan revealed small bowel obstruction, likely caused by right ovarian metastasis with direct infiltration into the small intestine (Figure 3C). She underwent the operation after the decompression of the dilated intestine. Based on the operative findings, the enlarged right ovary, measuring 6 × 4 × 3 cm, infiltrated the bowel wall 220 cm distal to the ligament of Treitz, and the small intestine was retracted and bent at the site of infiltration into the bowel wall, which caused the obstruction. Consequently, right oophorectomy and partial resection of the small intestine were performed (Figure 4A). We did not resect the locally recurrent tumor and the left ovary because her general condition or activities of daily living had decreased since the primary surgery. The ovarian tumor did not grow into the lumen in the resected specimen. Although the tumor actually contained ovary‐specific tissues such as ovarian stromal cells pathologically, most of that showed well‐differentiated tubular adenocarcinoma (tub1). Immunohistochemically, the ovarian tumor was negative for cytokeratin 7 (CK7), and positive for CK20 and caudal‐related homeobox 2 (CDX2). The immunohistochemistry findings of the resected specimen of the primary lesion were similar to those of the ovarian tumor (Figure 5). Thus, the final diagnosis was ovarian metastasis from cecal cancer. Although chemotherapy (S‐1) was resumed for residual local recurrence, liver and lung metastases occurred 12 months after the second surgery (47 months after the primary surgery). After that, her general condition gradually became deteriorated, and she died 53 months after the primary surgery.

## 3 DISCUSSION

As a primary lesion of metastatic ovarian tumors, the percentage of colorectal cancer is comparatively low (1.6%–6.4%), but it has increased recently.^2^, ^3^ Some studies have reported that the 5‐year survival rate for ovarian metastasis from colorectal cancer ranged from 0% to 29.1%.^4^, ^5^, ^6^ Ueda et al. reported that the 3‐year survival rate was also low at 20%.^7^ Ovarian metastasis from colorectal cancer is associated with peritoneal dissemination and metastasis to organs other than the ovary. This contributes to its poor prognosis. In particular, peritoneal dissemination is an essential prognostic factor. Miller et al. reported that the median survival time of colorectal cancer patients with ovarian metastasis and peritoneal dissemination was 10 months, whereas in the absence of peritoneal dissemination, the median survival time was 25 months.^8^ Patients with solitary ovarian metastasis reportedly had a good prognosis after oophorectomy. Tomiki et al. reported that the 5‐year survival rate in 10 patients with solitary ovarian metastasis was 67.5%.^9^, ^10^ Furthermore, McCormick reported that oophorectomy for patients with metastasis to organs, including the ovary, also contributed to prolonged survival.^11^ Chemotherapy for colorectal cancer reportedly had a lower response rate in patients with ovarian metastasis than those with metastasis to other organs, with a response rate of 0%–5% in patients with ovarian metastasis and 40%–60% in patients with metastasis to other organs.^12^, ^13^ Kim et al. reported that oophorectomy was the most effective treatment for ovarian metastasis from colorectal cancer.^14^ The above findings suggest that oophorectomy is significant in treating ovarian metastasis from colorectal cancer with or without metastasis to other organs.

On the contrary, from the viewpoint of symptom management, palliative surgery for ovarian metastasis from colorectal cancer is also performed. There are some cases of palliative resection for huge metastatic ovarian tumor which caused abdominal distention.^15^ In this case, the patient had a solitary ovarian metastasis in addition to local recurrence, which caused small bowel obstruction by direct infiltration into the small intestine. Thus, oophorectomy was unavoidable in relieving the condition. Even though we should have resected the locally recurrent tumor and the left ovary according to the oncological principles, we performed only right oophorectomy since her general condition had not been maintained. As a result, she could survived without a new recurrence for 12 months after the second surgery despite local recurrence and single‐agent chemotherapy. However, it is uncertain that only right oophorectomy contributed to achieve prolonged survival. If radical resection and/or intensive chemotherapy after the second surgery had been done, she might have followed a different clinical course.

To our best knowledge, a case of intestinal obstruction due to ovarian metastasis from colorectal cancer has not been reported previously. Since an oncologic emergency can suddenly occur like this case even if metastatic ovarian tumor is not huge, oophorectomy should be performed actively not only to improve the prognosis but also to prevent the deterioration of quality of life.

## 4 CONCLUSION

We reported a case of small bowel obstruction due to right ovarian metastasis after ileocecal resection for cecal cancer, which was treated surgically. It is obvious that oophorectomy for ovarian metastasis from colorectal cancer is necessary to achieve prolonged survival according to the oncological principles. However, an unexpected event related to metastatic ovarian tumor can occur like this case even if that is not huge. Therefore, oophorectomy should be also performed actively from the viewpoint of symptom management and maintaining quality of life.

## CONFLICT OF INTEREST

The authors declare that they have no conflict of interest.

## AUTHOR CONTRIBUTIONS

Naohiko Otsuka wrote the manuscript. Kimiyuki Shirayama supervised the writing of the manuscript. All authors read and approved the final manuscript.

## ETHICAL APPROVAL

This study was conducted in accordance with the 1964 Declaration of Helsinki and its later amendments.

## CONSENT

Written informed consent was obtained from the patient for publication of this case report and accompanying images.

## Data Availability

The data that support the findings of this study are available from the corresponding author upon reasonable request.
